# Fermat Principle, Ramsey Theory and Metamaterials

**DOI:** 10.3390/ma16247571

**Published:** 2023-12-09

**Authors:** Mark Frenkel, Shraga Shoval, Edward Bormashenko

**Affiliations:** 1 Chemical Engineering Department, Engineering Faculty, Ariel University, Ariel 407000, Israel; markfr@ariel.ac.il; 2 Department of Industrial Engineering and Management, Faculty of Engineering, Ariel University, Ariel 407000, Israel; shraga@ariel.ac.il

**Keywords:** Fermat principle, optical path, metamaterials, left-handed materials, graphs, Ramsey theory, Ramsey theorem, optical cycle

## Abstract

Reinterpretation of the Fermat principle governing the propagation of light in media within the Ramsey theory is suggested. Complete bi-colored graphs corresponding to light propagation in media are considered. The vertices of the graphs correspond to the points in real physical space in which the light sources or sensors are placed. Red links in the graphs correspond to the actual optical paths, emerging from the Fermat principle. A variety of optical events, such as refraction and reflection, may be involved in light propagation. Green links, in turn, denote the trial/virtual optical paths, which actually do not occur. The Ramsey theorem states that within the graph containing six points, inevitably, the actual or virtual optical cycle will be present. The implementation of the Ramsey theorem with regard to light propagation in metamaterials is discussed. The Fermat principle states that in metamaterials, a light ray, in going from point *S* to point *P*, must traverse an optical path length *L* that is stationary with respect to variations of this path. Thus, bi-colored graphs consisting of links corresponding to maxima or minima of the optical paths become possible. The graphs, comprising six vertices, will inevitably demonstrate optical cycles consisting of the mono-colored links corresponding to the maxima or minima of the optical path. The notion of the “inverse graph” is introduced and discussed. The total number of triangles in the “direct” (source) and “inverse” Ramsey optical graphs is the same. The applications of “Ramsey optics” are discussed, and an optical interpretation of the infinite Ramsey theorem is suggested.

## 1. Introduction

This paper presents the synthesis of the Fermat principle and Ramsey theory and reports the optical interpretation of the Fermat principle, applied to left-handed metamaterials. The Fermat principle (in its initial formulation, which was essentially corrected recently and will be discussed below) states that the actual path taken by a beam of light between two points is the one that is traversed in the least time [1]. This principle is one of the variational principles of physics and plays a fundamental, basic role in its structure (including the Maupertuis and Hamilton principles and the Hilbert variational principle for general relativity). The Fermat principle was formulated in 1662 by Pierre de Fermat, a French mathematician and lawyer but was anticipated nearly a millennium ago by the Arab scientist Ibn al-Haytham and inspired by the reflections of the Greek polymath Hero of Alexandria (Hρων o Aλεξανδρε’υς) on light almost two millennia ago [2,3]. The Fermat principle is more accurately and rigorously formulated with the use of the notion of the optical length/optical path length. The optical length/optical path length *L* between points, $P_{1}$ and $P_{2}$, is defined according to Equation (1):

(1) $$L=\int_{P_{1}}^{P_{2}}nds$$

where *n* is the refraction index of the medium, taken as a function of distance along the optical path.

The principle of Fermat asserts that the actual optical path of an optical ray between any two points, $P_{1}$ and $P_{2}$, is shorter than an optical length of any other curve that joins these points and that lies in a certain regular neighborhood of it [1,4]. Thus, the Fermat principle is often regarded as the principle of the least optical path. Following is Equation (2):

(2) $$nds=\frac{c}{v}vdt=cdt$$

and the substitution of Equation (2) into Equation (1) gives rise to Equation (3)

(3) $$L=c\int_{P_{1}}^{P_{2}}dt,$$

which immediately results in the principle of the least time, as was formulated by Pierre de Fermat. His principle states that the actual path between two points taken by a beam of light is the one that is traversed in the least time, which is weaker than the Principle of the Least Optical Length [1,4].

The development of metamaterials (or left-handed materials) led to the generalization of the Fermat principle [5,6,7,8]. Veselago predicted that electromagnetic plane waves in a medium having simultaneously negative electric permittivity ε and magnetic permeability μ would propagate in a direction opposite to that of the flow of energy [5,6,7,8]. This result follows not from the wave equation, which remains unchanged in the absence of sources, but rather from the individual Maxwell curl equations. In left-handed materials, vectors $\vec{k}$, $\vec{E}$ and $\vec{H}$ form a left-handed set, while in the usual materials (ε>0, μ>0), they form a right-handed set. Applications of left-handed materials include lenses, antennas and effective radio-cloaking [8,9]. In metamaterials, the Fermat principle is generalized as follows: a light beam, in going from point *S* to point *P*, must traverse an optical path length *L* that is stationary with respect to variations of this path [6].

We demonstrate in our paper how the Ramsey theory may be applied to the analysis of optical systems. The application of the Ramsey theory to optical systems employs the Fermat principle in which points in physical space are represented by the vertices of the graph and the optical paths represent the edges/links of the graph. The Ramsey theory, introduced by the British mathematician and philosopher Frank Plumpton Ramsey, is the field of combinatorics/graph theory that deals with a specific kind of mathematical structure, namely complete graphs. A graph is a mathematical structure comprising a set of objects in which some pairs of objects are in some sense “related” [10,11]. A complete graph is a graph in which each pair of graph vertices is connected by an edge/link. The typical problem considered by the Ramsey theory (as it was stated by Frank Ramsey) is the so-called “party problem”, which predicts the minimum number $R\left(m,n\right)$ of participants that must be gathered together in a room (each of whom is either a friend or a stranger to the others) so that at least *m* of the participants will be acquainted with each other or at least *n* of them will not be familiar with each other [12,13,14,15,16]. In this case, $R\left(m,n\right)$ is known as the Ramsey number [12,13,14,15,16]. Consider the particular formulation of the party problem: “What is the smallest number of people in a gathering, every two of whom are either friends or strangers, that will guarantee that there are either three mutual friends or three mutual strangers in the gathering”? In this particular case, $R\left(3,3\right)=6.$ A classical tenet of the Ramsey theory states that if some mathematical structure/graph is separated into many finite subparts, then one of the subparts must contain a substructure/graph of the given type. Aphoristically speaking, the Ramsey theory is the study of the preservation of properties under the set partitions [16].

The rigorous mathematical statement of the Ramsey theorem is formulated as follows: For any k+1≥3 positive integers *t*, $n_{1},\;n_{2},\;\ldots n_{k},$ there exists a positive integer *N* such that if each of the *t*-element subsets of the set $\left\{1,2,\ldots N\right\}$ is colored with one of the *k* colors 1, 2, …, *k*, then for some integer *i* with 1≤i≤k, there is a subset *S* of $\left\{1,2,\ldots N\right\}$ containing $n_{i}$ elements such that every *t*-element subset of *S* is colored *I* [16]. Frank Plumpton Ramsey, Paul Erdős and Ronald Lewis Graham significantly contributed to the development of the Ramsey theory [15,17,18].

Somewhat surprisingly, the physical and engineering applications of the Ramsey theory are still scarce. For example, the Ramsey approach was successfully applied to the theories of communication and decision making [19]. A Ramsey theory of financial distortions was reported recently [20]. The dynamic Ramsey theory of mechanical systems forming complete graphs in its relation to the analysis of vibrations of cyclic compounds was addressed [21]. Successful application of the Ramsey theory for the formulation of axiomatic thermodynamics was reported [22]. We demonstrate for the first time the application of the Ramsey theory in optical systems. We also demonstrate how the Ramsey approach may be reshaped for optical metamaterials [5,6,7,8,9,23,24,25,26].

## 2. Results

### 2.1. Ramsey Approach to Optical Systems: From Real Optical System to a Graph

**Figure 1:**
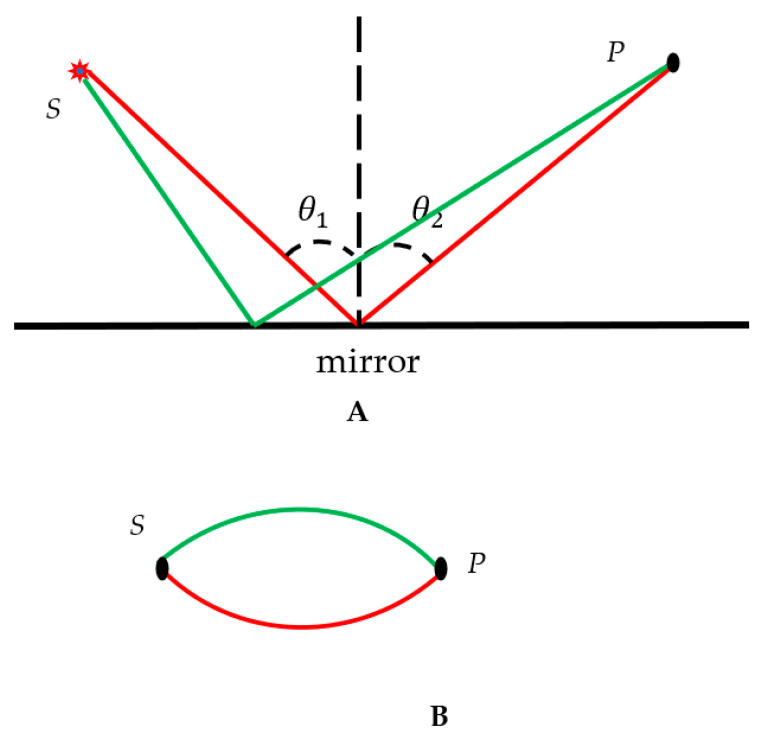
Conversion of the reflection experiment into the bicolor scheme is depicted. (**A**) The system consists of a source, placed at point *S*, and a sensor and an ideal mirror, located at point *P*; thus, $\theta_{1}=\theta_{2}$. Two kinds of optical paths are depicted. The red trajectory corresponds to the actual optical path; the green trajectory corresponds to the trial/virtual optical path. (**B**) Bi-colored sketch, corresponding to the reflection experiment, is shown.

The Ramsey theory considers complete graphs in which the vertices are connected with at least two kinds of edges/links (multicolored Ramsey graphs were also addressed [27]). Let us explain in detail how we applied the Ramsey theory to optical systems. We considered the simplest system consisting of a source, located at point *S*, and a mirror and a sensor located at point *P*, as depicted in Figure 1A. The mirror is supposed to be ideal; thus, the usual law of light reflection works [1], and the angle of reflection equals the angle of incidence $\theta_{1}=\theta_{2}$, as shown in Figure 1A.

Two kinds of optical paths of the beam generated by the source, located at point *S*, were possible: the actual path depicted by the red line in Figure 1A, for which the reflection law $\theta_{1}=\theta_{2}$ is true, and the trial/test/virtual path depicted by the green line in Figure 1A. The actual/red optical path emerged from the Fermat principle [1]. The use of the trial pathways is the usual procedure that is followed within the variational principles of physics, such as the Hamilton principle and the Fermat principle [1]. We propose now the following mathematical procedure to convert the real optical experiment depicted in Figure 1A into the scheme shown in Figure 1B. The vertices in the graph in Figure 1B correspond to the actual points *S* and *P* in the source/mirror/sensor experiment, shown in Figure 1A, and the red and green edges/links correspond to the actual/trial optical paths presented in Figure 1A. According to the principle of the reversibility of light, if the path of the light is reversed after suffering a number of reflections and refractions, then it retraces its path [1]. Thus, vertices *S* and *P* may be replaced. It should be emphasized that the coloring scheme, presented in Figure 1B, is not a graph; in a graph, two vertices are connected by a unique edge/link, which is not necessarily represented by a straight line segment but instead may be schematically “skewed”. The coloring scheme that we introduced omits the real physical processes occurring within the physical system and represents only the actual and trial optical paths of light propagation.

**Figure 2:**
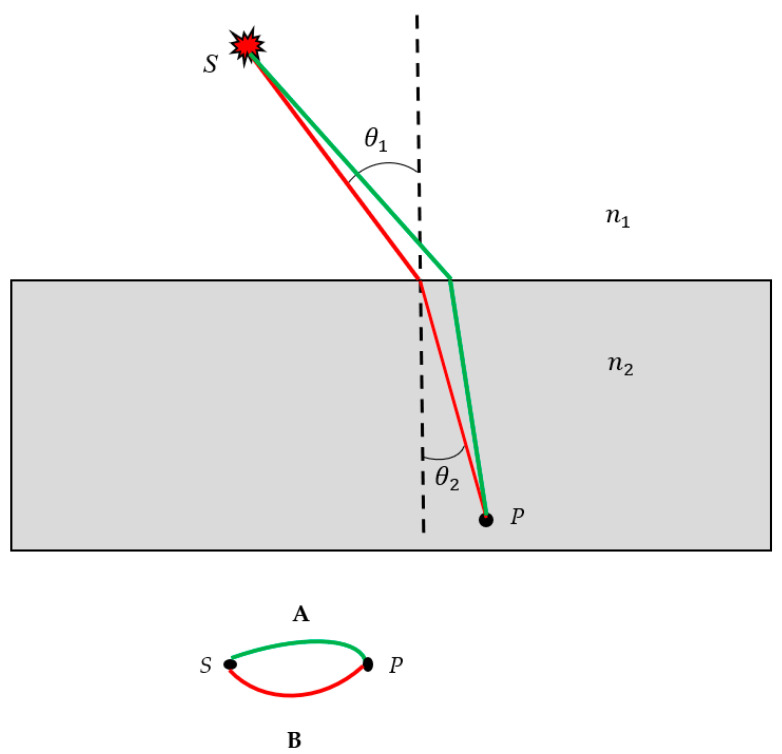
Bi-color sketch that emerged from the refraction experiment is depicted. (**A**) Light beam passed from the medium with the refraction index $n_{1}$ to the medium with the refraction index $n_{2}.$ The interrelation between the angles $\theta_{1}$ and $\theta_{2}$ is given by Snell’s law:$\;n_{1}sin\theta_{1}=n_{2}sin\theta_{2}$. Two kinds of optical paths are depicted. The red trajectory corresponds to the actual optical path; the green trajectory corresponds to the trial/virtual optical path. (**B**) Bi-colored scheme, corresponding to the refraction experiment, is shown.

Next, we applied the aforementioned procedure for the conversion of a real optical event to the optical scheme that emerged from the refraction experiment. When the light forming a ray moves from one medium to another—say from air to a glass slab—the incident ray changes direction at the boundary between the media; the ray is said to undergo refraction (see Figure 2A). Let the index of refraction of the medium with the incident ray be $n_{1}$ and that of the medium with the refracted ray be $n_{2}$. Every optical medium is characterized by a dimensionless number (refraction index); the difference between the indices at the interface between two media gives an indication of the light-bending ability of that interface. The angles that the incident and refracted rays make with the line normal to the boundary between the media are denoted $\theta_{1}$ and $\theta_{2}$ (the angles are shown in Figure 2A and the normal is depicted by the dashed line). The interrelation between the angles and the refraction indices is given by Equation (4):

(4) $$n_{1}sin\theta_{1}=n_{2}sin\theta_{2}.$$

This result, discovered by Willibord Snell in 1621, is known as a Snell’s law [1]. In an effort to restore historical justice, it should be mentioned that the refraction and reflection of light were first studied by Ḥasan Ibn al-Haytham (965–1040), a medieval mathematician, astronomer and physicist of the Islamic Golden Age from present-day Iraq [28]. Ibn al-Haytham was the first to correctly explain the theory of vision and to argue that vision occurs in the brain, pointing to observations that it is subjective and affected by personal experience [28]. He also developed the principle of least time for refraction, which would later become the Fermat principle, addressed in the Introduction section. The conversion of the refraction experiment into the bi-colored scheme is illustrated in Figure 2. The source was located at point *S* and the sensor was placed at point *P*. Two kinds of optical paths of the beam generated by the source, located at point *S*, were possible: the actual path, depicted by the red line in Figure 2A, for which the Snell/Ibn al-Haytham law ($n_{1}sin\theta_{1}=n_{2}sin\theta_{2}$) is true, and the trial/test/virtual path depicted by the green line in Figure 2A.

Again, according to the principle of the reversibility of light, the vertices *S* and *P* may be mutually replaced [1].

**Figure 3:**
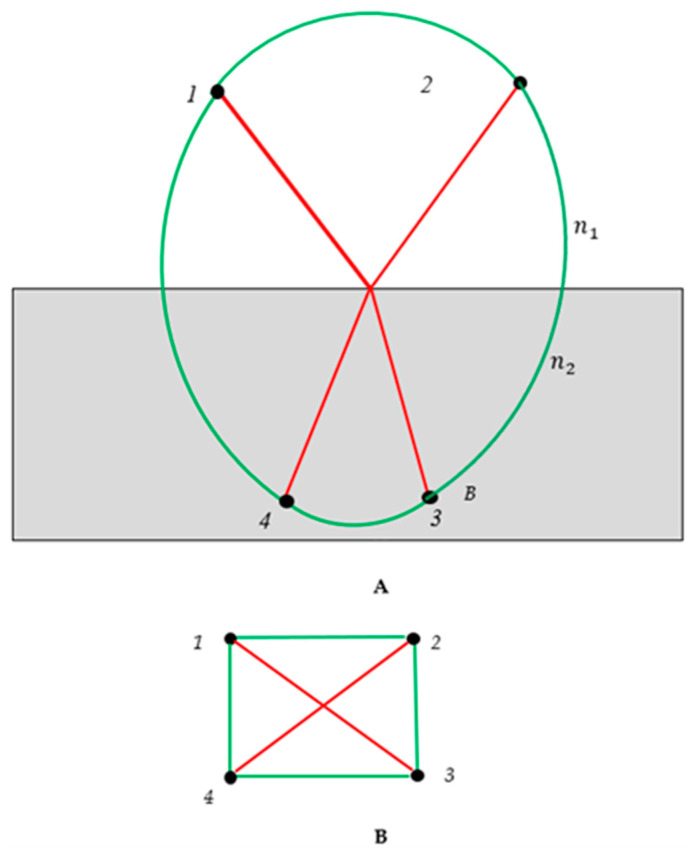
Formation of the optical graph emerging from the light refraction is demonstrated. (**A**) Refraction of the light generated by the sources located at points “1” and “2” is depicted. Sensors were placed at points “3” and “4”. Red lines correspond to the actual optical paths; green lines correspond to the trial optical paths, which actually do not occur. (**B**) Conversion of the optical experiment to the bi-colored, non-directed optical graph, which consisted of 4 vertices and 6 links, is depicted. No mono-colored triangle/cycle is recognized in the graph. This is possible due to the Ramsey theorem, $R\left(3,3\right)=6$.

Next, we describe the optical experiment in which light rays were generated by a pair of sources, located at points “1” and “2”, as shown in Figure 3A. Light was refracted at an interface that separated two media with refractive indices $n_{1}$ and $n_{2}$ (see Figure 3A). The light sources were placed at points “1” and “2”; the sensors were placed at points “3” and “4”. Again, we considered two kinds of optical paths in Figure 3A: the actual paths (colored with red), which are governed by Snell’s law, and the trial/virtual paths (shown with green links), which actually do not occur. Every pair of vertices is connected by a single link. Thus, the complete bi-colored graph, depicted in Figure 3B emerged (and it should be emphasized that Figure 3B depicts a graph). We call this graph the “optical graph”. This graph contains no mono-colored (fully red or green) triangles; in other words, no optical cycle is recognized in the graph. Indeed, according to the Ramsey theorem, it is possible to create a complete bi-colored graph in which no mono-colored triangles appear, and this is due to the $R\left(3,3\right)=6$ (in other words, we need at least six vertices in order to expect a mandatory appearance of the mono-colored triangle in the bi-colored graph; we recognize only four vertices in the optical graph, depicted in Figure 3B). Again, it should be emphasized that the optical graph depicted in Figure 3B is complete, bi-colored, and non-directed [10,11,12,13,14].

**Figure 4:**
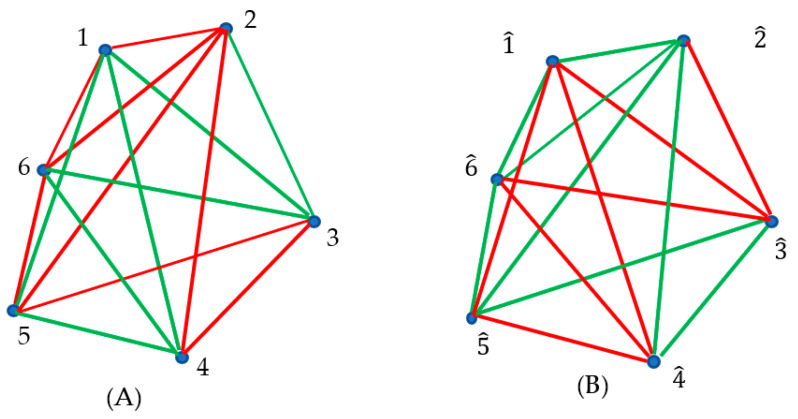
(**A**) Complete, bi-colored graph corresponding to the optical experiment in which light sources are located at the points labeled “1”, “2” and “3”. Red paths correspond to the actual optical paths; green paths correspond to the trial optical paths, which actually do not occur. The triangles “126” and “256” are mono-colored and consist of red edges only. The mono-colored triangles represent actual optical cycles. (**B**) The inverse graph emerging from the graph shown in (**A**) is depicted; triangles $\left(\hat{1}\hat{2}\hat{6}\right)$ and $\left(\hat{2}\hat{5}\hat{6}\right)$ are monochromatic and represent the optical cycles, which do not occur.

Next, we outline the optical experiment in which we placed three sources at the points “1”, “2” and “3”; sensors were placed at points “4”, “5” and “6”. Again, we considered two kinds of optical paths: actual paths (we do not specify the physics of the light propagation), colored with red, and the trial/virtual paths, which actually do not occur, shown with green links in Figure 4A. Each pair of vertices is connected by a unique link/optical path. Thus, the optical complete, non-directed, bi-colored graph, depicted in Figure 4A, emerged.

It should be emphasized that the coloring of the links is non-transitive. Indeed, consider the propagation of the light according to the red/actual paths 1→2, followed by actual propagation 2→3. In this case, points “1” and “3” may be connected by red/actual or green/virtual paths/links. This fact is very important in light of the application of the Ramsey theorem for the analysis of complete, bi-colored graphs. The values of transitive Ramsey numbers calculated for transitive graphs are different from those established for non-transitive graphs [29].

According to the Ramsey theorem, at least one monochromic triangle/cycle should appear within the graph, shown in Figure 4A, due to the fact that the Ramsey number is $R\left(3,3\right)=6$. Indeed, the triangles “126” and “256” consist of the red edges, and they correspond to actual optical cycles (see Figure 4A). This result will be true for any optical experiment, represented by the bi-colored, complete, undirected graph comprising six vertices; namely, at least one green or red optical cycle will appear. Thus, we recognized two so-called “optical cycles” appearing in Figure 4A. Recently, optical cycles have attracted the attention of investigators in the fields of photonics and nano-photonics [30,31,32]. Regrettably, it is impossible to predict what kind of mono-colored triangle, red/actual or green/virtual, will appear in the optical graphs; the Ramsey theory has no tools for such a prediction, and therefore, it is impossible to predict what kind of cycles (actual or virtual) will be present in the graphs. This is, of course, a weak point of the Ramsey approach.

Next, we introduce the notion of the inverse bi-color Ramsey graphs, generated by the source graph; we replaced red links appearing in the source graph with green ones, and vice versa, as shown in panel (B) of Figure 4. In other words, the actual optical paths were replaced with the trial/virtual ones, and vice versa. The inverse optical graph is a complete graph. The vertices of this optical graph are denoted $\left(\hat{1},\ldots \hat{6}\right)$ in panel (B) of Figure 4. We call such a Ramsey network the “inverse graph”. Obviously, introducing an inverse Ramsey network is possible for any complete source graph, and in particular, for the graphs representing optical experiments. According to the Ramsey theorem, both the source and inverse optical graphs, arising from six vertices, contain at least one monochromatic triangle. Thus, actual or virtual optical cycles will be present in both the source and inverse optical graphs. Indeed, we recognize the red monochromatic triangles $\left(126\right)$ and $\left(256\right)$ in panel (A), and correspondingly the green triangles $\left(\hat{1}\hat{2}\hat{6}\right)$ and $\left(\hat{2}\hat{5}\hat{6}\right)$ in panel (B) of Figure 4. Green triangles represent virtual optical cycles, which actually do not occur.

It is noteworthy that the total number of triangles in the “direct” (source) and “inverse” Ramsey optical graphs is the same, thus yielding the conservation law:

(5) $$\zeta =t_{r}+t_{g}={\hat{t}}_{r}+{\hat{t}}_{g},$$

where $t_{r}\;and\;t_{g}$ are the numbers of red and green triangles in the source graph; correspondingly, ${\hat{t}}_{r}\;and\;{\hat{t}}_{g}$ are the numbers of red and green triangles in the inverse graph. Equation (5) represents the “conservation law” for the Ramsey complete network that consists of six elements. It is noteworthy that direct and inverse graphs form the Abelian (commutative group) in which the inversion of the color of the link is taken as an operation.

### 2.2. Ramsey Approach to Metamaterials

**Figure 5:**
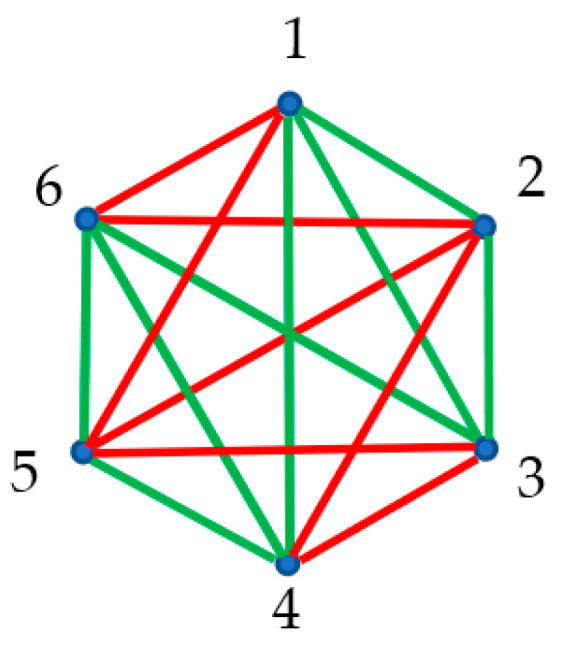
Complete optical graph corresponding to propagation of light in the metamaterial. The vertices of the graph correspond to the points in physical space in which sources or sensors are placed. Links correspond to the actual optical rays. Red links correspond to the optical paths for which the function $L\left(s\right)$ demonstrates a maximum; green links correspond to the optical paths for which the function $L\left(s\right)$ demonstrates a minimum. Triangles “456” and “123” are mono-colored (green).

The alternative application of the Ramsey approach is possible for light propagating in metamaterials (left-handed media [5,6]). We already mentioned that in the metamaterials, the Fermat principle should be generalized as follows: a light ray, in going from point *S* to point *P*, must traverse an optical path length *L* that is stationary with respect to variations of this path [6]. By a stationary value of the function $L\left(s\right)$ (see Equation (1)), we mean one for which the slope of $L\left(s\right)$ versus *s* is zero or, equivalently, where the function $L\left(s\right)$ has a maximum, minimum or point of inflection with a horizontal tangent [1,6]. The kind of extremum (maximum, minimum or inflection point) depends on the actual values of the refraction index of the medium [1,6]. Thus, one more optical interpretation of the Ramsey theory becomes possible in metamaterials. For the sake of simplicity, we exclude an exotic situation in which the optical path corresponds to the inflection point of the function $L\left(s\right).$ Thus, bi-colored, complete graphs, similar to those depicted in Figure 5, become possible.

Next, we considered the graph that consists of six vertices, which correspond to the points in physical space in which sources or sensors are placed. All of the links of the graph correspond to the actual optical pathways. We assume that red links correspond to the optical paths for which the function $L\left(s\right)$ demonstrates a maximum and that green links, in turn, correspond to the optical paths for which the function $L\left(s\right)$ demonstrates a minimum. Thus, the complete, undirected optical graph, such as that depicted in Figure 5, emerge. According to the Ramsey theorem, this complete graph inevitably contains at least one mono-colored triangle. Indeed, triangles “456” and “123” are mono-colored (green). Thus, two optical cycles that consist of the optical pathways “456” and “123”, for which $L\left(s\right)$ is minimal, are observed in this optical experiment [30,31,32]. Now, these pathways are actual optical paths.

Again, the inverse optical graph may be defined according to the procedure introduced in Section 2.1, and the conservation law described by Equation (5) holds true.

### 2.3. Optical Interpretation of the Infinite Ramsey Theorem

**Figure 6:**
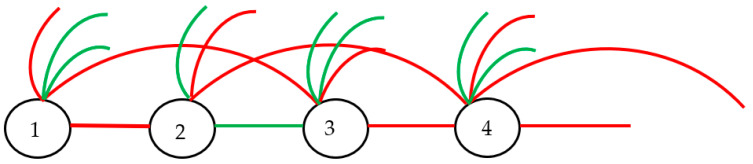
Infinite Ramsey theorem is illustrated for two-colored infinite graphs: any two-colored $K_{N}$ must contain a red or green monochromatic countably infinite complete subgraph.

Until now, we have discussed finite graphs. In this section, we will address infinite complete graphs, infinite Ramsey theory and their optical interpretation, which gives rise to infinite optical graphs. The infinite Ramsey theory states that if the complete graph of a countably infinite set is colored with finitely many colors, then there is an infinitely monochromatic clique. A clique is a subset of vertices of an undirected graph such that every two distinct vertices in the clique are adjacent. We will illustrate the infinite Ramsey theorem with an understandable example [11,12,13,14,15]: Imagine joining every pair of positive integers with a line, as shown in Figure 6. Every pair of positive integers is joined by a line/link. Let us denote the emerging graph as $K_{\infty }$. Now we color each link either red or green; thus, we build a complete two-colored graph. The infinite two-color Ramsey theorem states that no matter how we color the edges in $\;K_{\infty },$ it will always be possible to find infinitely many points that are all connected by the same color. In other words, consider $K_{\infty }$, which is a complete graph whose vertex set is countably infinite; every two-colored $K_{\infty }$ must contain a countably infinite monochromatic complete graph. The infinite Ramsey theorem for multicolored graphs states that if we color each edge of an infinite, complete graph with one out of finitely many prescribed colors, then there is an infinite, complete monochromatic subgraph. That is, an infinite set of vertices such that all edges among them have the same color.

A more general version of the infinite Ramsey theorem states that if we split an infinite object with a certain regularity property (such as a set containing arbitrary long arithmetical progressions) into two parts, one infinite part will exhibit this property, too [12].

Next, we will provide an optical interpretation of the infinite Ramsey theory. Consider an infinite number of points in a physical space, numbered “1”, “2”, “3”,…, with sources or sensors located at the points. Every point is joined by a link corresponding to the optical event/optical path: red links represent the actual optical paths corresponding to light propagation between the connected points, whereas green links correspond to the trial/virtual optical paths, which do not actually occur. Two points are connected by a single link. Thus, the complete, undirected optical graph, similar to that depicted in Figure 6, emerged. According to the infinite Ramsey theorem, an infinite, complete, monochromatic subgraph (either green or red) will appear in the graph; in other words, an infinite monochromatic clique will be present in the graph. Thus, the actual or trial/virtual optical path connecting all of the points will be present in the graph. According to the principle of the reversibility of light, the addressed infinite bi-colored optical graph will be non-directional. The generalization of the infinite Ramsey theorem for metamaterials is straightforward (see Section 2.2) if we assume that the red links correspond to the optical paths for which the function $L\left(s\right)$ demonstrates a maximum and that the green links, in turn, correspond to the optical paths for which the function $L\left(s\right)$ demonstrates a minimum.

## 3. Discussion

This paper presents a synthesis of the Fermat principle and the Ramsey theory. The variational principles of physics and, in particular, the Fermat principle, which describes the least optical path length, classify all of the possible paths connecting the points in physical space as actual or virtual. Actual paths correspond to trajectories of light propagation, whereas virtual or trial paths correspond to light ray trajectories that do not occur. We define that physical points are “acquainted with one with another” when they are connected by an actual optical path using sources and sensors and are “strangers” when they are connected by virtual/trial optical paths. Thus, the introduction of the Ramsey approach for the analysis of light propagation in complex media becomes possible. With this approach, we are completely distracted from the peculiarities of optical events taking place during the propagation of a light ray from one point to another; we consider only the demands of the Fermat principle, which may be fulfilled or violated. Thus, we conclude that in the “optical graph” containing six vertices, the appearance of at least one actual or virtual optical cycle is inevitable due to the fact that $R\left(3,3\right)=6$.

Let us discuss the potential applications of the suggested Ramsey analysis of optical systems. One of the fields in which the Ramsey analysis is expected to be useful is two-wavelength digital holographic microscopy [33]. In this method, red and green lights are employed for the illumination of an object, and the generated holograms are recorded simultaneously by a color CCD camera [33]. With this method, a pair of virtual detectors are introduced, one of which is located in front of the real detector, and the other is behind it [34]. This makes the application of the suggested Ramsey analysis straightforward. If the studied optical scheme contains less than six vertices, no optical cycle may appear in the scheme. If the scheme contains six vertices (which are sources or sensors), at least one optical cycle will inevitably appear in the scheme, as is discussed in Section 2.2. Regrettably, the Ramsey theorem does not prescribe what kind of optical cycle, actual or virtual, will be present, and this is a weak point of the Ramsey analysis.

Another field of optics in which the Ramsey analysis is expected to be useful is the field of fiber optical communications, in particular, the analysis of so-called *p*-cycles. In optical communications, “a lightpath is an optical connection from a source node to a destination node which is carried over a wavelength on each intermediate link. At intermediate nodes, the lightpaths are routed and switched from one link to another link” [35]. Fiber optics demonstrates numerous advantages; it is a category of cable-based technologies in which optical fibers are either buried under the ground, attached to poles or placed at the bottom of ocean. In all of these cases, the optical connection is surprisingly vulnerable to cable cuts [35]. One of the suggested solutions to this problem is the creation of virtual protection cycles, abbreviated as *p*-cycles [35]. The basic idea of a *p*-cycle is the creation of virtual protection paths by utilizing the concept of fully pre-cross-connected linear segments, resulting in the formation of a complete optical graph [35]. In this case, the ideas of the Ramsey theory are also applicable.

One more field of optics in which the Ramsey approach may be useful is optical cycling, which is used for the cooling of atoms, thereby enabling new pathways in ultra-cold physics [36,37]. Using a technique called Doppler cooling, a sample of atoms is irradiated with laser light whose frequency is tuned just below an atomic resonance [36,37]. Each atom preferentially absorbs photons that are blue-shifted into resonance—that is, the ones that oppose the atom’s motion [36,37]. The atom then reradiates the light in a random direction and returns to its ground state. Repeating that optical cycle some tens of thousands of times can cool the atomic sample to below 1 mK [36,37]. In this case, the Ramsey complete graph is introduced as follows: vertices denote the quantum levels, red links correspond to the actual quantum transitions, and green links, in turn, correspond to the forbidden quantum transitions [36,37]. Thus, in the graph consisting of six vertices, monochromatic cycles will inevitably appear. Again, it is impossible to predict what kind of cycles will emerge: actual or virtual.

## 4. Conclusions

The Ramsey theory provides an abstract framework for the analysis of a broad diversity of events/facts/states interconnected by various relations, forming a complete graph. In the simplest case of bi-colored graphs, the vertices may be considered as “friends” or “strangers”. Vertices may represent interacting particles or thermodynamic states [22], and the links on the graph represent at least two kinds of interrelations between the vertices (such as attraction or repulsion between interacting particles [38]). We demonstrated how “optical Ramsey graphs” may be introduced for convential optical media and metamaterials. For this purpose, we involved the Fermat principle of the least optical path lengths and its generalization for metamaterials. This principle divided all of the possible paths connecting two points, where light sources and sensors were located, into two classes: the actual paths/”friends” and the trial paths/”strangers”. The vertices/points were connected by a single link/optical path. This classification gave rise to a bi-colored, complete, non-directional graph, which represented real optical systems. Optical pathways in this graph connected the vertices, which represented the points in a real physical space. According to the principle of the reversibility of light, the complete bi-colored graph will be non-directional. Thus, the application of the Ramsey theorem became possible. For example, in the graph consisting of six vertices, at least one monochromatic optical cycle (actual or trial) was present. Regrettably, the Ramsey theory did not predict what kind of monochromatic cycle, actual or trial, appeared in the graph, and this is a weak point of the Ramsey approach. We introduced the notion of the “inverse optical graph”, generated using the original source optical graph. Regarding the bi-colored, complete optical graph, we replaced the actual optical paths (red links) with the trial optical pathways (green links) and vice versa; this procedure gave rise to the inverse optical graph. The total number of triangles in the “direct” (source) and “inverse” Ramsey optical graphs ζ was the same, thus yielding the conservation law $\zeta =t_{r}+t_{g}={\hat{t}}_{r}+{\hat{t}}_{g}$, where $t_{r}\;and\;t_{g}$ were the numbers of red and green triangles in the source optical graph and ${\hat{t}}_{r}\;and\;{\hat{t}}_{g}$ were the numbers of red and green triangles in the inverse optical graph.

The Ramsey theory may be fruitful in situations where the “optical cycles” emerge, such as two-wavelength digital holographic microscopy, optical communication (*p*-cycles) and the optical cooling of atoms.

An alternative interpretation of the Ramsey approach became possible for light propagating in metamaterials (left-handed media). In metamaterials, the Fermat principle was generalized as follows: a light ray, in going from point *S* to point *P*, must traverse an optical path length $L\left(s\right)$ that is stationary with respect to variations of this path *s.* We assumed that the red links corresponded to the optical paths for which the function $L\left(s\right)$ demonstrated a maximum and that the green links, in turn, corresponded to the optical paths for which the function $L\left(s\right)$ demonstrated a minimum (we assume that the function $L\left(s\right)$ did not demonstrate inflection points; this assumption kept the binary paradigm of analysis). Thus, the complete bi-colored optical graph emerged. According to the Ramsey theorem, the complete bi-colored graph, consisting of six vertices, inevitably contains at least one mono-colored triangle. Thus, optical cycles with a minimum of three vertices were observed in the optical experiment that we performed herein; these pathways were actual optical paths.

We introduced the notion of the infinite optical graph, consisting of an infinite number of points the physical space, which were numbered “1”, “2”, “3”,… Every point was joined to another point by a link corresponding to the optical event: the red link corresponded to actual optical paths representing the actual light propagation between the connected points and the green link corresponded to the trial/virtual optical path, which did not actually occur. Two points were connected with a single link. Thus, the complete, infinite, non-directional optical graph emerged. According to the infinite Ramsey theorem, an infinite, complete, monochromatic subgraph (either green or red) will appear in the graph. Thus, the actual or trial/virtual optical path connecting all of the points will be present in the graph.

## Data Availability

The data are contained within the article.

## References

[1] Hecht E. (2002). Section 4.5—Fermat’s Principle. Optics.

[2] Ceccarelli M. (2007). Distinguished Figures in Mechanism and Machine Science: Their Contributions and Legacies.

[3] Adamson P. (2016). Philosophy in the Islamic World: A History of Philosophy without Any Gaps.

[4] Born M., Wolf E. (1999). Principles of Optics.

[5] Veselago V.G. (2005). Some remarks regarding electrodynamics of materials with negative refraction. Appl. Phys. B.

[6] Veselago V.G. (2002). Formulating Fermat’s principle for light traveling in negative refraction materials. Physics-Uspekhi.

[7] Smith D.R., Kroll N.K. (2000). Negative Refractive Index in Left-Handed Materials. Phys. Rev. Lett..

[8] Shalaev V. (2007). Optical negative-index metamaterials. Nat. Photon.

[9] Pokrovsky A.L., Efros A.L. (2003). Lens based on the use of left-handed materials. Appl. Opt..

[10] Wilson R.J. (1996). Introduction to Graph Theory.

[11] Trudeau R.J. (1993). Introduction to Graph Theory.

[12] Katz M., Reimann J. (2018). An Introduction to Ramsey Theory: Fast Functions, Infinity, and Metamathematics, Student Mathematical Library.

[13] Graham R.L., Spencer J.H. (1990). Ramsey Theory. Sci. Am..

[14] Li Y., Lin Q. (2020). Elementary Methods of the Graph Theory, Applied Mathematical Sciences.

[15] Graham R., Butler S. (2015). Rudiments of Ramsey Theory.

[16] Landman B.M., Robertson A. (2004). Ramsey Theory on the Integers.

[17] Chartrand G., Zhang P. (2021). New directions in Ramsey theory. Discret. Math. Lett..

[18] Hoffman P. (1998). The Man Who Loved Only Numbers: The Story of Paul Erdős and the Search for Mathematical Truth.

[19] Roberts F.S. (1984). Applications of Ramsey theory. Discret. Appl. Math..

[20] Bassetto M., Cui W. (2021). A Ramsey Theory of Financial Distortions.

[21] Shvalb N., Frenkel M., Shoval S., Bormashenko E. (2023). Dynamic Ramsey Theory of Mechanical Systems Forming a Complete Graph and Vibrations of Cyclic Compounds. Dynamics.

[22] Shvalb N., Frenkel M., Shoval S., Bormashenko E. (2023). Ramsey theory and thermodynamics. Heliyon.

[23] Kar S. (2023). Metamaterials and Metasurfaces: Basics and Trends.

[24] Cai W., Shalaev V. (2010). Optical Metamaterials: Fundamentals and Applications.

[25] Engheta N., Ziolkowski R.W. (2006). Metamaterials: Physics and Engineering Explorations.

[26] Pendry J.B., Schurig D., Smith D.R. (2006). Controlling electromagnetic fields. Science.

[27] Dudek A., Prałat P. (2017). On some Multicolor Ramsey Properties of Random Graphs. SIAM J. Discret. Math..

[28] Masic I. (2008). Ibn al-Haitham--father of optics and describer of vision theory. Medical Arhiv..

[29] Choudum S.A., Ponnusamy B. (1999). Ramsey numbers for transitive tournaments. Discret. Math..

[30] Nehra R., Sekine R., Ledezma L., Guo Q., Gray R.M. (2022). Few-cycle vacuum squeezing in nanophotonics. Science.

[31] Leblond H., Mihalache D. (2013). Models of few optical cycle solitons beyond the slowly varying envelope approximation. Phys. Rep..

[32] Shuman E.S., Barry J.F., Glenn D.R., DeMill D. (2009). Radiative Force from Optical Cycling on a Diatomic Molecule. Phys. Rev. Lett..

[33] Gao P., Yao B., Rupp R., Min J., Guo R., Ma B., Zheng J., Lei M., Yan S., Dan D. (2012). Autofocusing based on wavelength dependence of diffraction in two-wavelength digital holographic microscopy. Opt. Lett..

[34] Wen Y., Wang H., Anand A., Qu W., Cheng H., Dong Z., Wu Y. (2019). A fast autofocus method based on virtual differential optical path in digital holography: Theory and applications. Opt. Lasers Eng..

[35] Kiaei M.S., Assi C. (2009). A Survey on the *p*-Cycle Protection Method. IEEE Commun. Surv. Tutor..

[36] Mitra D., Lasner Z.D., Zhu G.-Z., Dickerson C.E., Augenbraun B.L., Bailey A.D., Alexandrova A.N., Campbell W.C., Caram J.R., Hudson E.R. (2022). Pathway toward Optical Cycling and Laser Cooling of Functionalized Arenes. J. Phys. Chem. Lett..

[37] Hofsäss S., Doppelbauer M., Wright S.C., Kray S., Sartakov B.G., P´erez-Ríos J., Meijer G., Truppe S. (2021). Optical cycling of AlF molecules. New J. Phys..

[38] Shvalb N., Frenkel M., Shoval S., Bormashenko E. (2023). Universe as a Graph (Ramsey Approach to Analysis of Physical Systems). World J. Phys..

